# Association between changes in gene signatures expression and disease activity among patients with systemic lupus erythematosus

**DOI:** 10.1186/s12920-018-0468-1

**Published:** 2019-01-09

**Authors:** Michelle Petri, Wei Fu, Ann Ranger, Norm Allaire, Patrick Cullen, Laurence S. Magder, Yuji Zhang

**Affiliations:** 1 Johns Hopkins University School of Medicine, Baltimore, MD USA; 2 Unum Therapeutics, Cambridge, MA USA; 3 Biogen, Cambridge, MA USA; 4 University of Maryland School of Medicine, Baltimore, MD USA

**Keywords:** Systemic lupus erythematosus, Interferon, SLE disease activity index, SLE activity

## Abstract

**Background:**

We assessed the stability of BAFF, interferon, plasma cell and LDG neutrophil gene expression signatures over time, and whether changes in expression coincided with changes in SLE disease activity.

**Methods:**

Two hundred forty-three patients with SLE were evaluated for disease activity, serological parameters and peripheral blood gene signatures in clinic visits (2 or more per patient) that occurred between 2009 and 2012. Levels of the BAFF gene transcript, plasma cell signature, Interferon (IFN) signature and the low density granulocytes (LDG)-associated neutrophil gene signature were assessed in PAX-gene-preserved peripheral blood by global microarray. The stability of repeated measures of gene expression was quantified using intra-class correlation coefficients. SLE disease activity was measured using the Physicians Global Assessment and the SELENA-SLEDAI index and its components. Using a mixed effects regression model we assessed: 1) the association between a patient’s average gene signature expression over time and disease activity, and 2) the association between a patient’s changes in gene expression over time and changes in disease activity.

**Results:**

Gene expression signatures showed more within-person stability than systolic blood pressure. The IFN signature exhibited the most stability. Patients with high levels of BAFF and IFN transcripts tended to have significantly higher levels of musculoskeletal disease, skin disease, anti-dsDNA, and erythrocyte sedimentation rate, and lower levels of complement. However, changes in BAFF or IFN gene signatures were not associated with changes in disease activity. Similar associations were seen between the LDG gene signature and disease activity. However, when LDG increased, complement tended to increase. Patients with high levels of plasma cell gene signature tended to have higher levels of anti-dsDNA and lower levels of complement. However, unlike the other gene signatures, changes in plasma cell gene signature significantly coincided with changes in anti-dsDNA and complement.

**Conclusions:**

The gene expression signatures were relatively stable within patients over time. BAFF and interferon gene expression were markers of patients with generally higher disease activity, but changes in these gene signatures did not coincide with changes in disease activity. Plasma Cell gene signature expression tracked with the traditional SLE serologic markers of anti-dsDNA and complement.

## Background

Multiple gene expression signatures have been identified in systemic lupus erythematosus (SLE), including interferon (IFN) [1–3], B-cell activating factor (BAFF) [4], low-density granulocytes (LDG) [5], and plasma cell (PC) [6]. The natural history of these gene signatures is not yet fully understood, as studies of sensitivity to change are few.

IFN-stimulated genes are up-regulated in more than half of SLE patients [7, 8]. However, the potential of the IFN-alpha gene signature to predict change in disease activity over time is uncertain. Landolt-Marticorena et al. showed a lack of association between changes in the IFN-alpha signature and longitudinal changes in disease activity in SLE [9]. Petri et al. found little change in the IFN-response scores (calculated based on the expression of three IFNα-regulated genes) over time within individual patients [10]. However, Chiche et al. recently identified IFN signatures that are variable over time in single patients [11]. Interestingly, they also found a variable IFN signature, not restricted to IFN-alpha, but also driven by IFN-beta and gamma.

BAFF, also known as the B lymphocyte stimulator (BLyS), is tied to the pathogenesis of SLE. It is expressed as a transmembrane protein on monocytes, macrophages, and monocyte-derived dendritic cells and critical for B-cell growth and survival [12]. Elevated circulating levels of BAFF were found in SLE patients, correlate with increased total IgG and autoantibody (particularly anti-dsDNA) levels [13] and associated with increased disease activity (as measured by the SLE-Disease Activity Index; SLEDAI) [14]. Although the serum BAFF level was generally found to be correlated with SLEDAI scores in SLE patients in cross-sectional comparisons, it did not always correlate with changes in disease activity over time [14, 15]. Collins and colleagues found that BAFF mRNA levels from peripheral blood leukocytes were correlated with disease activity measured by the SLEDAI, better than BAFF serum protein levels [4]. Zollars et al. showed that BAFF gene expression level was strongly associated with clinical and serologic SLE activity on the same day and predictive of clinical activity over the next year [16].

Neutrophils and plasma cells have an important role in the induction of autoimmune responses and organ damage in SLE [17, 18]. SLE-derived neutrophils can trigger increased cell death and enhanced extracellular trap formation [19]. A distinct type of abnormal subset of neutrophils seen in SLE, LDG, has been associated with vascular damage and skin involvement [17]. Neutrophil-specific genes are highly expressed in peripheral blood mononuclear cell (PBMC) in SLE [20, 21]. Recently, Jourde-Chiche identified a neutrophil signature significantly associated with lupus nephritis [22].

Streicher et al. found that the plasma cell signature was increased in blood from SLE patients compared to blood from healthy donors and also significantly correlated with SLE disease activity [23]. Petri et al. [24] found elevated plasma cell gene signature was associated with leukopenia, anti-Ro, anti-La and the lupus anticoagulant, but not with the same day clinical activity by Physician Global Assessment (PGA) or SLEDAI.

In this study, we assessed the stability of these gene signatures over time in patients with SLE. In addition we addressed two related questions: 1) Do those individuals who have high levels of a gene signature also tend to have high levels of specific types of activity, and 2) Does a change in gene signature levels coincide with a change in specific types of disease activity.

## Methods

### Patients, ethics and study design

The study protocol for SPARE (Study of biological Pathways, disease Activity and REsponse markers in patients with systemic lupus Erythematosus) was approved by the Johns Hopkins University School of Medicine Institutional Review Board (Study number NA_00039294). All patients enrolled from the Hopkins Lupus Cohort provided written informed consent. SLE was defined according to the revised American College of Rheumatology classification criteria or the Systemic Lupus International Collaborating Clinics (SLICC) classification criteria [25–27]. Gene signatures were measured for more than one visit for 243 patients in the SPARE study, from September of 2009 to June of 2012.

### Patient assessment

Patients were treated according to standard clinical practice. At entry into the Hopkins Lupus Cohort, the medical history was reviewed and recorded. All patients were evaluated by the same physician at entry and all subsequent cohort visits (Dr. Michelle Petri). To assess disease activity, the Safety of Estrogens in Lupus Erythematosus: National Assessment (SELENA) version of the Systemic Lupus Erythematosus Disease Activity Index (SLEDAI) [28, 29] as well as the PGA [30] were completed at each visit. C3, C4, anti-dsDNA, complete blood cell count and urinalysis were performed at every visit. Visits were scheduled quarterly, or more often if required for disease activity.

### Gene signature expression

Levels of the IFN signature, BAFF gene transcript, the LDG-associated Neutrophil gene signature and Plasma Cell signature were assessed in PAX gene-preserved peripheral blood by global microarray. All gene signatures were defined log-transformed normalized expression values from the Affymetrix chip. Gene signatures are defined as follows: three IFN-annotated modules (M1.2, M3.4, and M5.12) were found by Chiche et al. to be strongly up-regulated in SLE patients [11]. BAFF was a single gene product defined on the Affymetrix chip as (223501_PM_at). The neutrophil signature was defined as an average value of the signal from the following genes (DEFA4, CEACAM8, BPI, OLFM4, LTF, LCN2, CEACAM6, MMP8). These specific genes were chosen as they were found to be enriched in the granulopoesis gene set of Bennett et al. [20], the neutrophil set of Berry et al. [31] and the definition of the low density granulocytes in Villanueva et al. [21]. The reduced set of eight genes was found to have good internal correlation. The plasma cell signature was defined by the following genes IGJ: immunoglobulin J chain and TXNDC5: thioredoxin domain containing protein 5.

### Statistical analysis

For multi-gene signatures, the geometric mean of RNA expression of each component gene was calculated. Bland-Altman plots were constructed to visualize the stability of gene signature within individual patients. The stability of repeated measures of gene expression was quantified using intra-class correlation coefficients (ICC). ICC was calculated by comparing the variability of different measurements of the same subject to the total variation across all measurements and all subjects. To provide a frame of reference we also computed the ICC for systolic blood pressure and weight.

SLE disease activity was measured using the PGA and the SELENA-SLEDAI index. Subtypes of SLE disease activity was measured using SLEDAI component scores without multiplication by the weighting factor. To assess the association between gene signature levels and disease activity levels we used a regression model developed by Neuhaus and Kalbfleish [32]. This model partitions the association between a time-varying predictor (a gene signature) and a time-varying outcome (a disease activity level) into two components: a “between person” component and a “within-person” component. The between person component quantifies the degree to which people with generally higher levels of a gene signature tend to also have generally higher levels of the disease outcome. This is identified by including a person’s average gene signature level across all clinic visits into a model as a predictor of disease activity outcome at each clinic visit. The “within-person” component quantifies the degree to which changes in gene signature levels in a person coincide with changes in disease activity. This is identified by including a variable representing the difference between a person’s gene signature level at a specific clinic visit and a person’s average gene expression as a predictor of disease activity at that specific clinic visit.

### Data availability

The microarray data set has been deposited in the NCBI Gene Expression Omnibus (GEO) database (GEO accession number GSE45291 and GSE121239).

## Results

Two hundred forty-three patients with SLE were evaluated for disease activity, serological parameters and IFN-alpha, BAFF, neutrophils and plasma cells gene expression level in a series of clinic visits over a 2-year period. 143 SLE patients contributed two visits, 40 patients contributed three visits and 60 patients contributed four or more visits. The patients were 92% female with a median age of 47 years (Interquartile range 37–55). They were 58% Caucasian American, 35% African American, and 7% of other race/ethnic groups. The average SELENA-SLEDAI at baseline was 2.4 (SD = 2.6) with a range from 0 to 12.

Of the 243 patients in the analysis, 62 had SLEDAI score of 0 for all follow-up visits. In addition, 54 additional patients had positive SLEDAI, but no changes throughout follow-up. These stable patients did not contribute to the “within patient” analysis. Almost all the remaining patients had a change of SLEDAI of two points or more during the follow-up.

**Fig. 1:**
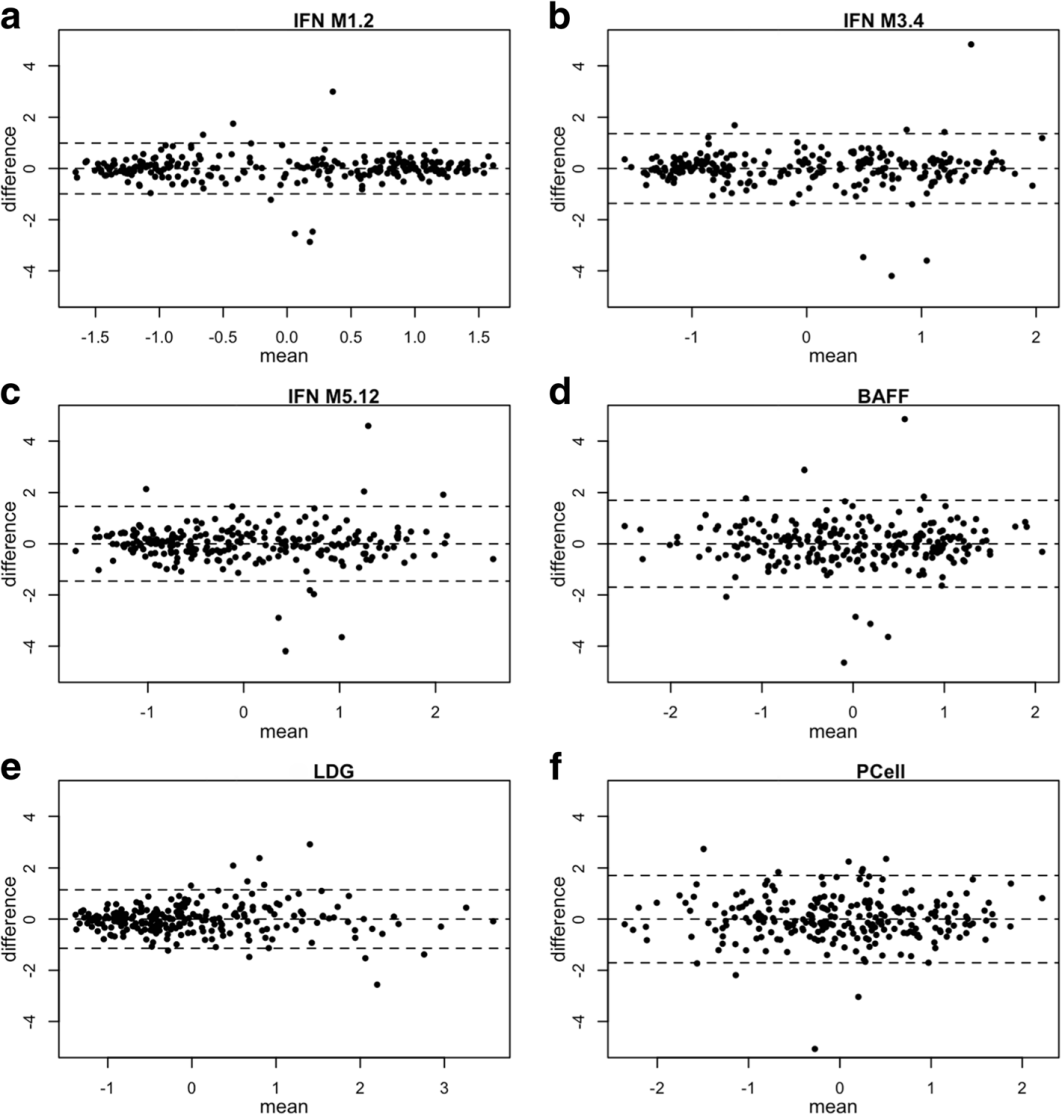
Bland-Altman Plot of Six Gene Signatures at First Two Visits in SPARE Study. Each dot represents one patient, the x axis shows the mean of gene signatures at the patient’s first two visits, the y axis shows the difference between the measures of gene signatures for those two visits, the middle dash-line indicates overall mean of difference, and the other two dash-lines are 95% upper and lower limits of agreement. **a**. IFN Module 1.2 signature, **b**. IFN Module 3.4 signature, **c**. IFN Module 5.12 signature, **d**. BAFF signature, **e**. LDG signature, **f**. Plasma Cell signature

Figure 1 shows Bland-Altman plots for different gene signatures, comparing baseline visit and the first follow-up visit in the SPARE study. For 77% of the patients, the time separation between the first and second visit was between 60 and 120 days, but the range was from 4 to 487 days. Patients were shown to be clustered into two groups by IFN M1.2 gene signatures, which is mainly associated with IFN-alpha. Patients with low level of IFN M1.2 gene signatures were scattered between 95% limits of agreement in IFN-alpha gene signatures; while patients with high level of IFN-alpha gene signature mostly had little difference between baseline visit and follow-up visits. IFN M3.4 and IFN M5.12, which are associated with IFN-beta and IFN-gamma, did not show similar patterns as IFN M1.2 gene signature. Similarly, for the other three gene signatures (BAFF, LDG and plasma cell), differences were distributed evenly regardless of mean.

Table 1 shows the Intra-class correlation for the different biomarkers. In addition, to provide some points of reference, the table shows the Intra-class correlation for systolic blood pressure, weight and SLEDAI. Weight was consistent between two visits with Intra-class correlation of 0.98. All gene signature scores were more stable than systolic blood pressure and disease activity, with higher Intra-class correlation.

**Table 1:** Within-patient Intraclass Correlation Coefficients (ICC) for Gene Expression Signatures, Disease Activity, Systolic Blood Pressure, and Weight

Gene Expression	ICC	95% Confidence Interval
IFN module 1.2	0.88	0.85,0.9
IFN module 3.4	0.79	0.75,0.82
IFN module 5.12	0.75	0.7,0.79
BAFF Signature	0.66	0.61,0.72
Neutrophil (LDG) Signature	0.81	0.77,0.84
Plasma Cell Signature	0.61	0.54,0.66
SELENA SLEDAI	0.35	0.26,0.44
Physician’s Global Assessment	0.43	0.34,0.5
Weight	0.98	0.98,0.98
Systolic Blood Pressure	0.52	0.45,0.59

Tables 2 and 3 show the between-person and within-person associations between gene signatures and various types of SLE disease activity. There was a strong between-person association between the IFN and BAFF gene signatures and traditional serologic indicators of SLE (anti-dsDNA, low complement) (Table 2). That is, those with a large average levels of IFN or BAFF expression tend to have higher anti-dsDNA and lower complement (*p* < 0.0001 for most of those relationships). Similar relationships were seen between average expression of these genes and ESR (Table 2). In contrast, however, there was no strong evidence of a within-person association between those gene signatures and anti-dsDNA, low complement, or erythrocyte sedimentation rate (ESR) (Table 3). That is, for example, in an individual, changes in those signatures over time were not associated with changes in anti-dsDNA, complement, or ESR. There was also evidence of a between-person association between IFN or BAFF gene signatures and both musculoskeletal and skin disease activity (Table 2, *p*-values in the range of 0.0016 to 0.062). Again, there was no corresponding within-person association for these relationships. In fact, there was no strong evidence of a within-person association between IFN or BAFF gene signatures and any type of SLE disease activity (Table 3).

**Table 2:** “Between Patient” Analysis: Association between a patient’s average value of the gene signature and their average value of each disease activity measure^a,b^

	IFN M1.2		IFN M3.4		IFN M5.12		BAFF		LDG		Plasma Cells	
	Estimate (95% CI)	*p* value	Estimate (95% CI)	*p* value	Estimate (95% CI)	*p* value	Estimate (95% CI)	*p* value	Estimate (95% CI)	*p* value	Estimate (95% CI)	*p* value
PGA	0.05 (− 0.02, 0.12)	0.16	0.07 (− 0.00, 0.14)	0.064	0.08 (0.01, 0.16)	0.026	0.06 (− 0.02, 0.13)	0.15	0.06 (− 0.02, 0.13)	0.12	− 0.00 (− 0.08, 0.07)	0.90
SLEDAI	**0.94** **(0.68, 1.20)**	**< 0.0001**	**1.00** **(0.73, 1.26)**	**< 0.0001**	**1.06** **(0.80, 1.33)**	**< 0.0001**	**0.92** **(0.64, 1.21)**	**< 0.0001**	**0.62** **(0.32, 0.91)**	**< 0.0001**	0.11 (− 0.21, 0.42)	0.50
SLEDAI Vascular	0.01 (− 0.01, 0.02)	0.50	0.01 (−0.01, 0.02)	0.81	−0.00 (− 0.02, 0.02)	0.92	0.00 (− 0.01, 0.02)	0.58	0.00 (− 0.02, 0.02)	0.98	0.01 (− 0.01, 0.03)	0.19
SLEDAI MUSCULOSKELETAL	0.02 (−0.00, 0.04)	0.062	**0.03** **(0.01, 0.05)**	**0.015**	**0.03** **(0.01, 0.05)**	**0.0038**	**0.03** **(0.01, 0.05)**	**0.013**	**0.03** **(0.01, 0.05)**	**0.0099**	0.00 (−0.02, 0.03)	0.74
SLEDAI Renal	0.01 (−0.03, 0.04)	0.70	0.01 (−0.03, 0.04)	0.61	0.02 (−0.02, 0.05)	0.35	0.01 (−0.03, 0.05)	0.57	0.02 (− 0.02, 0.05)	0.39	−0.03 (− 0.07, 0.01)	0.095
SLEDAI Skin	**0.08** **(0.03, 0.14)**	**0.0042**	**0.08** **(0.03, 0.14)**	**0.0045**	**0.09** **(0.03, 0.15)**	**0.0021**	**0.10** **(0.04, 0.16)**	**0.0016**	0.05 (− 0.00, 0.11)	0.069	−0.02 (− 0.08, 0.04)	0.57
SLEDAI Hematologic	0.01 (− 0.01, 0.04)	0.23	0.02 (− 0.01, 0.04)	0.17	0.01 (−0.01, 0.04)	0.35	0.00 (−0.02),0.03)	0.71	−0.02 (− 0.04, 0.01)	0.19	0.01 (− 0.02, 0.03)	0.44
SLEDAI Immunology)	**0.30** **(0.23, 0.37)**	**< 0.0001**	**0.32** **(0.24, 0.39)**	**< 0.0001**	**0.34** **(0.27, 0.42)**	**< 0.0001**	**0.27** **(−.18, 0.35)**	**< 0.0001**	**0.17** **(0.09, 0.25)**	**< 0.0001**	0.06 (− 0.03, 0.15)	0.18
Urine Protein Creatinine Ratio	−0.06 (− 0.16, 0.05)	0.29	− 0.06 (− 0.17, 0.05)	0.32	− 0.04 (− 0.15, 0.07)	0.50	− 0.09 (− 0.20, 0.03)	0.14	0.02 (− 0.09, 0.13)	0.74	− 0.05 (− 0.17, 0.07)	0.40
Log Anti-dsDNA	**0.83** **(0.60, 1.05)**	**< 0.0001**	**0.88** **(0.65, 1.11)**	**< 0.0001**	**0.95** **(0.72, 1.18)**	**< 0.0001**	**0.69** **(0.44, 0.95)**	**< 0.0001**	**0.54** **(0.30, 0.78)**	**< 0.0001**	**0.32** **(0.05, 0.58)**	**0.019**
C3	**−11.9** **(−16.2, −7.6)**	**< 0.0001**	**−13.0** **(−17.4, −8.6)**	**< 0.0001**	**− 12.7** **(− 17.1, − 8.3)**	**< 0.0001**	**−10.8** **(− 15.5, −6.0)**	**< 0.0001**	−1.7 (−6.3, 2.9)	0.46	**−6.4** **(− 11.3, − 1.6)**	**0.0099**
C4	**−2.28** **(−3.44, − 1.13)**	**0.0001**	**−2.32** **(−3.51, − 1.14)**	**0.0001**	**− 2.26** **(− 3.46, − 1.06)**	**0.0002**	**−1.88** **(− 3.15, − 0.61)**	**0.0038**	− 0.72 (− 1.91, 0.48)	0.24	−1.05 (− 2.32, 0.23)	0.11
ESR	**8.79** **(5.86, 11.72)**	**< 0.0001**	**9.17** **(6.18, 12.16)**	**< 0.0001**	**9.95** **(6.95, 12.95)**	**< 0.0001**	**8.18** **(4.93, 11.44)**	**< 0.0001**	**4.82** **(1.69, 7.96)**	**0.0026**	2.78 (− 0.59, 6.14)	0.11

^a^The entries in the table represent the expected difference in average disease activity measure per 1 standard deviation difference in average level of the gene signature. Bolded entries had *p*-values less than 0.05

^b^All the associations are adjusted for prednisone use, age, sex and race

**Table 3:** “Within Patient” analysis: Association between changes in gene signature and changes in disease activity between two visits from the same person^a,b^

	IFN M1.2		IFN M3.4		IFN M5.12		BAFF		LDG		Plasma Cell	
	Estimate (95% CI)	*p* value	Estimate (95% CI)	*p* value	Estimate (95% CI)	*p* value	Estimate (95%CI)	*p* value	Estimate (95% CI)	*p* value	Estimate (95% CI)	*p* value
PGA	0.11 (− 0.03, 0.25)	0.12	0.09 (− 0.02, 0.19)	0.10	0.05 (− 0.04, 0.15)	0.28	0.02 (− 0.06, 0.10)	0.64	− 0.06 (− 0.17, 0.05)	0.29	0.07 (− 0.01, 0.15)	0.074
SLEDAI	0.35 (− 0.26, 0.97)	0.26	0.31 (−0.15, 0.77)	0.18	0.29 (−0.13, 0,71)	0.17	0.16 (−0.20, 0.53)	0.38	−0.06 (− 0.54, 0.42)	0.80	**0.45** **(0.10, 0.80)**	**0.011**
SLEDAI Vascular	0.02 (−0.01, 0.06)	0.25	0.01 (−0.01, 0.04)	0.34	0.01 (−0.02, 0.03)	0.45	0.01 (−0.01, 0.03)	0.33	−0.00 (− 0.03, 0.03)	0.97	0.01 (− 0.01, 0.03)	0.27
SLEDAI MUSCULOSKELETAL	0.02 (−0.03, 0.07)	0.36	0.02 (−0.02, 0.06)	0.34	0.01 (−0.02, 0.05)	0.41	0.01 (−0.02, 0.04)	0.58	−0.01 (− 0.05, 0.03)	0.65	0.01 (− 0.02, 0.04)	0.65
SLEDAI Renal	0.03 (−0.06, 0.11)	0.54	0.03 (−0.03, 0.09)	0.36	0.05 (−0.01, 0.11)	0.078	0.02 (−0.03, 0.07)	0.36	0.03 (−0.03, 0.10)	0.33	0.02 (−0.03, 0.06)	0.54
SLEDAI Skin	−0.03 (− 0.11, 0.06)	0.53	− 0.00 (− 0.07, 0.06)	0.88	−0.02 (− 0.07, 0.04)	0.60	−0.04 (− 0.09, 0.01)	0.15	0.03 (− 0.03, 0.10)	0.31	0.02 (− 0.02, 0.07)	0.31
SLEDAI Hematologic	**−0.04** **(− 0.07, − 0.01)**	**0.0095**	**−0.03** **(− 0.05, − 0.01)**	**0.0096**	**−0.03** **(− 0.05, − 0.01)**	**0.014**	**−0.02** **(− 0.04, − 0.00)**	**0.028**	**−0.03** **(− 0.06, − 0.01)**	**0.0060**	0.01 (− 0.02, 0.03)	0.36
SLEDAI Immunology	0.00 (− 0.08, 0.09)	0.92	0.00 (− 0.06, 0.06)	0.96	−0.02 (− 0.08, 0.03)	0.42	0.00 (− 0.05, 0.05)	0.97	**−0.08** **(− 0.14, − 0.01)**	**0.026**	**0.09** **(0.04, 0.14)**	**0.0002**
Urine Protein Creatinine Ratio	0.06 (−0.07, 0.19)	0.36	0.05 (−0.05, 0.15)	0.32	0.05 (−0.04, 0.13)	0.31	0.06 (−0.02, 0.14)	0.12	−0.04 (− 0.14, 0.06)	0.44	0.05 (− 0.02, 0.13)	0.17
Log Anti-dsDNA	0.05 (−0.16, 0.25)	0.65	0.05 (−0.10, 0.20)	0.52	−0.01 (− 0.15, 0.13)	0.94	0.03 (− 0.09, 0.15)	0.65	−0.05 (− 0.21, 0.11)	0.56	**0.20** **(0.08, 0.32)**	**0.0008**
C3	1.28 (− 3.56, 6.11)	0.60	1.99 (− 1.66, 5.63)	0.28	2.57 (−0.75, 5.88)	0.13	−0.20 (− 3.07, 2.67)	0.89	**5.71** **(1.90, 9.51)**	**0.0034**	**−3.60** **(− 6.38, − 0.81)**	**0.011**
C4	0.73 (−0.35, 1.82)	0.18	0.52 (−0.30, 1.34)	0.21	0.57 (−0.18, 1.31)	0.14	0.18 (−0.47, 0.83)	0.58	**1.05** **(0.19, 1.90)**	**0.017**	**−1.39** **(−2.01, −0.77)**	**< 0.0001**
ESR	2.19 (− 1.17, 5.55)	0.20	2.25 (−0.28, 4.78)	0.082	**2.43** **(0.13, 4.73)**	**0.039**	−0.14 (−2.15, 1.87)	0.89	2.05 (−0.63, 4.73)	0.13	**3.75** **(1.81, 5.70)**	**0.0002**

^a^The entries in the table represent the expected change in disease activity measure per 1 standard deviation change in the gene signature. Bolded enteries had *p*-values less than 0.05

^b^All the associations are adjusted for prednisone use, age, sex and race

Similar to IFN and BAFF gene signatures, there was a strong between-person association between the LDG gene signature and the anti-dsDNA, ESR, musculoskeletal, and skin disease activity. However, in contrast to BAFF and IFN gene signatures, there was no evidence of a between-person relationship between LDG gene signature and low complement. Interestingly, there was an opposite within-person relationship between the LDG gene signature and low complement: when a person’s LDG gene signature went up, complement tended to go up (Table 3, *p* = 0.0034 for C3).

Similar to IFN and BAFF gene signatures, there was evidence of a between-person association between the Plasma Cell gene signature and traditional SLE serologies (anti-dsDNA and low complement). Unlike the other gene signatures, there was not a strong between-person association between Plasma Cell and either skin or musculoskeletal activity. However, there was a strong within-person relationship between the Plasma Cell signature and anti-dsDNA, C4, and ESR. That is, when Plasma Cell signatures increased, anti-dsDNA and ESR increased and C4 went down (*P* < 0.001 for all these associations, Table 3).

Changes in each gene signatures (except the plasma cell signature) were inversely related to the hematologic component of SLEDAI (*p* values ranging from 0.014 to 0.0060) (Table 3). That is, decreases in a patient’s gene signature levels were associated with an increased risk of leukopenia or thrombocytopenia.

## Discussion

In this study, we observed that the within-person stability (by Intra-class correlation) of the different gene expression signatures exceeded the stability of systolic blood pressure and disease activity (by SLEDAI) (Table 1). Of the gene signatures, the IFN M1.2 signature exhibited the most stability. As shown in Fig. 1, the distribution of change in our 6 gene signatures was centered on zero, with the BAFF and Plasma Cell gene signatures exhibiting slightly more variance than the 3 IFN and Neutrophil (LDG) gene signatures. Regarding the variance of the IFN Modules recently defined by Chiche et al. [11], Module 1.2 was the most stable (Intra-class correlation of 0.86), followed by IFN modules 3.4 and 5.12 (Intra-class correlation of 0.75, 0.7, respectively). Other previous studies [9–11] have also showed IFN scores do not significantly vary over time.

In our study, we found those with high levels of each IFN gene signature module tended to have higher values of skin and muscuoloskeletal disease activity as well as worse values of classic SLE serologic markers (anti-dsDNA and complement). However, within a person, changes in these gene signatures were not associated with changes in disease activity. These findings are consistent with two previous studies that looked separately at between-patient and within-patient effects for the IFN gene signature only [9, 10]. This suggests that the IFN gene signature is a marker of a certain type of patient, or the gene signature reflects chronic conditions that affect the outcome, but is less acutely associated with the pathogenic process.

Similarly, we found those with generally higher levels of BAFF gene expression tended to have higher levels of musculoskeletal, skin, and immunologic SLE disease activity. Using the same patients [16] we previously showed that a single measure of BAFF gene expression level was associated with clinical and serological SLE activity on the same day and predictive of clinical activity over the next year. However, in the present study, we found no relationship between changes in BAFF gene expression and changes in disease activity. This null finding is consistent with several previous studies of the relationship between changes in serum levels of BAFF and flares [14] or other changes in disease activity [15]. However, Carter et al. [33] showed that changes in serum BAFF levels did correlate positively with changes in anti-dsDNA antibody levels during relapse or remission after B Cell Depletion Therapy in SLE.

The LDG gene signature was found to be weakly associated with serological SLE activity in our study. Midgley et al. [34] observed increased expression in LDG neutrophils in juvenile-onset SLE patients, which correlated with anti-dsDNA antibody concentration and scores of disease activity. We found that the LDG gene signature was not associated with C3 and C4, which indicates that LDG gene signature could have different regulatory pathways, compared to the IFN and BAFF gene signatures. We also observed some evidence that those with higher levels of LDG gene signature expression tended to have more skin activity (*p* = 0.069) consistent with the observation by Denny et al. that elevated levels of LDG were associated with skin involvement [17].

The strongest “within-person” association we found was between the plasma cell gene signature and low C4. An increase of 1 standard deviation of the plasma cell gene signature was associated with a decline of 1.39 mg/dL in serum C4 (*p* < 0.0001).

This study has limitations. Firstly, whole blood gene-expression analysis does not permit identification of cell-specific components of the gene signature. On the other hand, it allows easy sample collection and preparation which are fundamental elements for clinical applicability. Secondly, the gene signature measurements were performed in two different batches at different times. A potential batch effect could affect the analysis. We measured 87 samples in both batches to minimize any batch effect.

In summary, the gene signatures that we studied were relatively stable within SLE patients over time. Consistent with previous studies, we found that individuals who tended to have high levels of BAFF or interferon gene signatures tended to have traditional serologic indicators of SLE (anti-dsDNA, low complement). They also tended to have higher average levels of musculoskeletal and skin disease activity. However, within individuals, changes in BAFF or interferon expression did not correlate with changes in disease activity. Similar findings were observed between the LDG gene signature and disease activity, however, increases in LDG coincided with increases in complement. Finally, there was a strong within-person correlation between changes in the Plasma Cell gene signature and changes in traditional SLE serologic indicators. Specifically, increases in plasma cell expression coincided with increases in anti-dsDNA and ESR, and decreases in complement. These relationships might provide some clues as to pathogenic processes or therapeutic targets.

## Conclusion

In this study, we assessed the stability of gene signatures over time in patients with SLE. The gene expression signatures were relatively stable. BAFF and interferon gene expression were markers of patients with generally higher disease activity, but changes in these gene signatures did not coincide with changes in disease activity. The plasma cell gene signature expression tracked with the traditional SLE serologic markers of anti-dsDNA and complement. These findings provide further insight into the utility of these signatures as predictors of the clinical course and of the pathogenic processes of SLE.

## Abbreviations

BAFF: B-cell activating factor
BLyS: B lymphocyte stimulator
ESR: Erythrocyte sedimentation rate
ICC: Intra-class correlation coefficients
IFN: Interferon
LDG: Low-density granulocytes
PBMC: Peripheral blood mononuclear cell
PC: Plasma Cell
PGA: Physician Global Assessment
SELENA: Safety of Estrogens in Lupus Erythematosus: National Assessment
SLE: Systemic lupus erythematosus
SLEDAI: SLE-Disease Activity Index
SLICC: Systemic Lupus International Collaborating Clinics
SPARE: Study of biological Pathways, disease Activity and REsponse markers in patients with systemic lupus Erythematosus
